# Experience Creates the Multisensory Transform in the Superior Colliculus

**DOI:** 10.3389/fnint.2020.00018

**Published:** 2020-04-21

**Authors:** Zhengyang Wang, Liping Yu, Jinghong Xu, Barry E. Stein, Benjamin A. Rowland

**Affiliations:** ^1^Department of Biomedical Engineering, Southern University of Science and Technology, Shenzhen, China; ^2^Key Laboratory of Brain Functional Genomics (Ministry of Education and Shanghai), School of Life Sciences, East China Normal University, Shanghai, China; ^3^Department of Neurobiology and Anatomy, Wake Forest School of Medicine, Winston-Salem, NC, United States

**Keywords:** cross-modal, development, timing, coactivation, enhancement, dark-rearing

## Abstract

Although the ability to integrate information across the senses is compromised in some individuals for unknown reasons, similar defects have been observed when animals are reared without multisensory experience. The experience-dependent development of multisensory integration has been studied most extensively using the visual-auditory neuron of the cat superior colliculus (SC) as a neural model. In the normally-developed adult, SC neurons react to concordant visual-auditory stimuli by integrating their inputs in real-time to produce non-linearly amplified multisensory responses. However, when prevented from gathering visual-auditory experience, their multisensory responses are no more robust than their responses to the individual component stimuli. The mechanisms operating in this defective state are poorly understood. Here we examined the responses of SC neurons in “naïve” (i.e., dark-reared) and “neurotypic” (i.e., normally-reared) animals on a millisecond-by-millisecond basis to determine whether multisensory experience changes the operation by which unisensory signals are converted into multisensory outputs (the “multisensory transform”), or whether it changes the dynamics of the unisensory inputs to that transform (e.g., their synchronization and/or alignment). The results reveal that the major impact of experience was on the multisensory transform itself. Whereas neurotypic multisensory responses exhibited non-linear amplification near their onset followed by linear amplification thereafter, the naive responses showed no integration in the initial phase of the response and a computation consistent with competition in its later phases. The results suggest that multisensory experience creates an entirely new computation by which convergent unisensory inputs are used cooperatively to enhance the physiological salience of cross-modal events and thereby facilitate normal perception and behavior.

## Introduction

A major issue of interest in sensory processing is how the brain develops the ability to use its different senses synergistically to enhance perception and behavior (Stein and Meredith, 1993; Murray and Wallace, 2012; Stein, 2012). This process of “multisensory integration” is ubiquitous, automatic, and effortless despite the complexity involved in coordinating the action of senses that have very different operational dynamics. However, this capability is neither innate, nor genetically pre-determined. Animal studies have suggested that the development of multisensory integration capabilities is shaped by multisensory experience, typically during early life, and that disrupting the acquisition of this experience, or the circuitry needed to properly process that experience, produces defective endpoints (see review by Stein et al., 2014). Anomalous development may help explain the compromised multisensory processing in a number of human populations, contributing to the sensory deficits in Autism Spectrum Disorder, Sensory Processing Disorder, Schizophrenia, and Dyslexia (Brett-Green et al., 2010; Williams et al., 2010; Brandwein et al., 2013; Stevenson et al., 2014, 2017; Beker et al., 2018).

The neural bases of multisensory development have been best documented in neurons of the superior colliculus (SC), a midbrain structure involved in detecting, localizing, and orienting toward environmental events (Stein and Meredith, 1993; Jiang et al., 2002; Burnett et al., 2004). In normally-developed adults, individual SC neurons generate amplified responses to spatiotemporally concordant visual-auditory stimuli (Meredith and Stein, 1986a; Wallace et al., 1998; Rowland et al., 2007), which are often derived from the same event (Parise et al., 2012; Kayser and Shams, 2015). This increases the physiological salience of the initiating event and the brain’s ability to organize appropriate behavioral responses to it. But in neonates, and animals reared in darkness, or with masking noise, or with exposure to random visual and auditory stimuli, SC responses to the same stimuli are not amplified, and often appear suppressed relative to their responses to the individual component stimuli (Wallace and Stein, 1997; Wallace et al., 2004; Royal et al., 2010; Yu et al., 2010, 2013b; Xu et al., 2012, 2014, 2015, 2017). The specific neuronal mechanisms by which multisensory experience changes the neural circuit to achieve normal functional outcomes are unknown.

One possibility is that multisensory experience changes the moment-by-moment operation that is used to transform unisensory inputs into a multisensory output; i.e., the “multisensory transform” (Miller et al., 2017). Thus, deficits in this process might reflect anomalies in forming the relevant synaptic configurations or other conformational properties of the underlying circuit. However, another possibility is that the multisensory experience acts to coordinate or calibrate the dynamics of the neuron’s converging unisensory inputs so that they are more amenable to integration (e.g., Engel et al., 2012). To assess these possibilities, we compared the response properties and moment-by-moment multisensory transform of neurons reared with normal multisensory experience to those reared in darkness. Understanding these relationships and dynamics is valuable both for understanding the development of the neural circuit underlying multisensory integration and for guiding the theory surrounding human perceptual anomalies in which multisensory processing appears to be compromised.

## Materials and Methods

### Animals

Data from two cohorts of mongrel cats (*Felis catus*) were evaluated: one set from neurotypic adults (*n* = 6, age > 1 year, weight = 2.5–5.0 kg) reared in a standard laboratory environment and one from animals (*n* = 5, age > 1 year, weight = 2.5–5.0 kg) reared in complete darkness (“dark-reared”). All animals were either obtained from a USDA-licensed commercial animal breeding facility (Liberty Research, Inc., Waverly, NY, United States) or born and raised in the Wake Forest Health Sciences housing facility. All procedures were carried out in accordance with the Guide for Care and Use of Laboratory Animals and approved IACUC protocols. Housing facilities were maintained by the local Animal Resources Program and were consistent with all local and federal housing guidelines. Other data obtained from some animals appear in previous publications (Perrault et al., 2005; Yu et al., 2010).

### Dark-Rearing

Animals were reared in a dark room that provided no visual or visual-auditory experience (see methods in Yu et al., 2010). A rotating cylinder prohibited all external light from entering this room, and animal husbandry was accomplished via night vision goggles. Litters were moved into this environment within days after birth while their eyes were still closed. Thereafter animals were raised to adulthood (approximately 1 year of age) before recording experiments were initiated.

### Recording Well-Implantation

Each animal was first anesthetized with a combination of ketamine hydrochloride (30 mg/kg, im) and acepromazine maleate (0.1 mg/kg, im) in its housing facility, its eyes were covered to preclude visual-auditory experience, and it was transported to the surgery suite in a covered carrier. It was then intubated and artificially respired to maintain end tidal CO_2_ level at 30 to 45 mmHg. Heart rate, blood pressure and spO_2_ level were monitored continuously and anesthesia was maintained with inhaled isoflurane (induction: 5%, maintenance: 1–3%). A craniotomy was made to provide access to the SC, a recording chamber was attached over that opening with screws and dental acrylic, and buprenorphine (0.005 mg/kg, im) and cefazolin (30 mg/kg, im) were provided twice each day for 3 days starting on the day of surgery.

### Electrophysiological Recording

Recording experiments began at least 1 week after well-implantation. In each experiment the animal was first anesthetized with ketamine/acepromazine and transported as described above. Animals were placed in a recumbent position and attached, via posts on the recording chamber, to a head stage on a recording platform. They were then intubated and paralyzed via pancuronium bromide (0.1 mg/kg, iv), respired and monitored as described above. Anesthesia, paralysis and hydration were maintained by continuous intravenous infusion of ketamine hydrochloride (5–10 mg kg^–1^ h^–1^) and pancuronium in lactated Ringer’s solution (2.4–5 ml/h). The optic disk was projected onto the tangent screen 44 cm from the eyes via reverse ophthalmoscopy, and the eyes were moistened with artificial tears. The eye contralateral to the recording site was fitted with a contact lens to focus the eye on a tangent screen, while the other was fitted with an opaque lens.

Visual stimuli were (10° × 2°) bars of light (13.67 cd/m^2^ against a background of 0.16 cd/m^2^) that were or flashed onto or moved (100°/s for 100 ms) across the tangent screen. Auditory stimuli were a brief burst (100 ms) of broad band noise (20–20,000 Hz) against an ambient background noise of 51.2–52.0 dB, delivered by 1 of 15 speakers mounted 15° apart on a metal hoop. Tungsten electrodes (tip diameter, 1–3 μm; impedance, 1–3 MΩ at 1 kHz) were driven into the intermediate/deep layers of the SC in search for single-unit activity. Neural activity was amplified and bandpass filtered between 500 and 5,000 Hz by a microelectrode amplifier (FHC). Single-unit spikes were isolated on the basis of spike height being at least three times that of background activity. Neurons were tested with a stimulus presented alone and in various combinations at multiple stimulus onsets varying from simultaneity to 100 ms (visual-before-auditory). Stimuli were presented at different locations within the overlapping regions of neurons’ visual and auditory receptive fields. Individual stimulus intensities were minimized in order to maximize the likelihood of observing multisensory enhancement (Meredith and Stein, 1986b).

### Response Windows, Magnitudes, Latencies, and Profiles

Response windows (defining latency and duration) for each stimulus condition were identified using a three-step geometric method described by Rowland et al. (2007). Overall response magnitudes were the trial-averaged number of impulses in this window minus the number expected based on the 500 ms pre-stimulus “spontaneous” window. Samples were only included in further analysis if the responses to the visual, auditory, and combined visual-auditory tests were significantly above zero (i.e., only “overt” multisensory neurons were examined, see, Yu et al., 2013a). Response latency was defined as the temporal delay of response window onset from stimulus onset (visual = LV, auditory = LA). Duration was the time between response onset and offset. Instantaneous firing rates were generated for each response by convolving the impulse raster with a Gaussian kernel (8 ms standard deviation) and averaging across trials. These firing rates were then corrected for baseline levels by subtracting the rate observed in the 500 ms window preceding the stimulus. Given variation in the visual and auditory response latencies across samples, it was necessary to identify for each sample a time point in each that could be used to align them according to when multisensory interactions would be expected to begin. This ‘Estimated Time of Convergence’ (ETOC) (see Miller et al., 2017) was calculated for a sample by summing the two unisensory response latencies (LV and LA) with the two stimulus onset delays (SV and SA), and finding the maximum.

(1) ETOC=max⁢(SV+LV,SA+LA)

### Metrics of Multisensory Enhancement

The metric of multisensory enhancement (ME) defined as the proportion increase of multisensory response magnitude (VA) over the largest unisensory response (visual = V, auditory = A), is a traditional quantitative measure of multisensory integration.

(2) $$\mathrm{ME}=\frac{\text{VA}-\max\left(V,A\right)}{\max\,\left(V,A\right)}$$

A sample was defined as “enhanced” if the multisensory response magnitude was significantly greater than the largest unisensory condition (independent 2-sample *t*-test), it was otherwise defined as “non-enhanced.” All statistical tests used an α criterion of 0.05.

Enhancement in the instantaneous multisensory response magnitude was also evaluated relative to the predictions of a statistical facilitation (aka “co-activation”) model. This model assumes that the visual and auditory channels independently activate the target multisensory neuron, but at each moment in time only the stronger determines the response. Because there is often substantial overlap in the distributions of the unisensory firing rates across trials, this prediction is often larger than the more robust of the unisensory responses but smaller than their sum. A bootstrap procedure was used to calculate its predictions at each moment in time by: (1) Calculating vectors for the trial-by-trial instantaneous firing rates for the unisensory visual (V) and auditory (A) responses, (2) Arranging a pairwise comparison between every visual and every auditory firing rate, and calculating the maximum of each pair to populate matrix M, where M_ij_ = max(V_i_, A_j_), and (3) For 100,000 repetitions, randomly drawing a sample from M equal in size to the number of multisensory trials and averaging it. Effect sizes and *p*-values for the actual mean multisensory firing rates were calculated using these distributions of predicted mean rates to determine significant deviations from statistical facilitation.

### Analyses of Unisensory Properties

Unisensory magnitudes, latencies, and durations were compared between groups using a 2-tailed independent *t*-tests. Unisensory imbalance (*UI*) was defined as the absolute difference in unisensory response magnitudes in proportion to their sum.

(3) $$\text{UI}=\frac{\left|\text{V}-\text{A}\right|}{V+A}$$

UI scores were compared between groups using a Wilcoxon rank-sum test. The temporal overlap between the unisensory responses was calculated using methods based on those described in Miller et al. (2015). For a pair of unisensory responses, the temporal overlap was the ratio between the areas under two curves as specified in (4) where *IFR*_Vk_ is the half wave-rectified visual instantaneous firing rate at the k_th_ millisecond in the response window.

(4) $$\mathrm{Overlap}=\frac{\sum_{\text{k}}\min\left({\mathrm{IFR}}_{\text{Vk}},{\mathrm{IFR}}_{\text{Ak}}\right)}{\sum_{\text{k}}\max\left({\mathrm{IFR}}_{\text{Vk}},{\mathrm{IFR}}_{\text{Ak}}\right)}$$

The impact of UI and temporal overlap on ME were determined using regression analyses. Best-fitting least-squares regression lines were fit to the relationships between ME vs. UI and temporal overlap in a multiple regression model. The slope and intercept parameters of these fits were statistically compared relative to zero, and across groups, using *t*-tests.

### Analyses of the Multisensory Transform

Millisecond-by-millisecond correlation analysis (pooling across neurons/samples after aligning by ETOC) was carried out between the instantaneous firing rate profile of the visual-auditory response and the summed profiles of the responses to the individually-presented visual and auditory components. The activity in selected time windows was extracted to summarize the temporal dynamics of multisensory response: an initial response window defined as [−20, 30 ms] around ETOC, and a later window following the end of the initial response until response offset. In addition, the temporal profiles of the multisensory responses were compared to the statistical facilitation predictions at each moment in time. Results presented in the text below indicate mean ± standard deviation unless otherwise indicated, results presented in the figures indicate mean ± standard error of the mean.

## Results

A total of 44 neurons in the “neurotypic” (i.e., normally-reared) cohort and 25 neurons in the dark-reared cohort were tested with a variety of effective visual and auditory stimuli presented alone (V, A) and in combination (VA). Multiple cross-modal tests in each neuron were conducted to ensure that the results were consist across variation in stimulus features. This yielded 161 VA samples from the neurotypic cohort (ME = 95 ± 51%) and 45 VA samples from the dark-reared cohort (mean ME = 6 ± 28%).

### Multisensory Transform

Neurotypic SC neurons synthesize their unisensory inputs into a multisensory output without wind-up or delay. This is apparent in the tight correlation between the dynamics of the instantaneous firing rate traces of the VA and summed V + A conditions after aligning based on stimulus onset (Miller et al., 2017). This finding was replicated here for the neurotypic sample, which showed a similarly tight correlation between these traces (0–200 ms after ETOC: mean *R*^2^ = 0.62, *p* < 0.001 at each millisecond). Interestingly, the dark-reared sample also showed a tight correlation between the multisensory and summed unisensory response dynamics (0–200 ms after ETOC: mean *R*^2^ = 0.67, *p* < 0.001 for all 1 ms steps) that was even stronger (*p* < 0.001, Wilcoxon signed-rank test). The correlation in the unisensory and multisensory dynamics observed in the dark-reared cohort suggests that, as in the neurotypic cohort, unisensory inputs are being continuously synthesized into multisensory outputs; i.e., both signals are received and processed by the target neuron.

**FIGURE 1:**
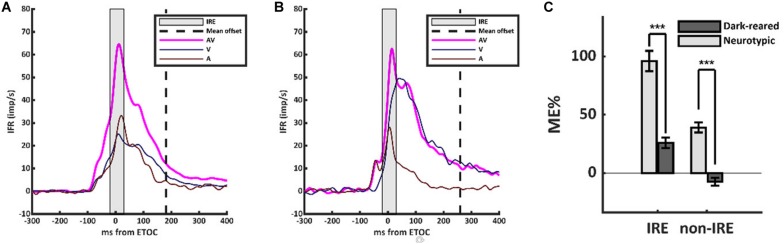
The multisensory transform did not form in the dark-reared cohort. **(A)** Population-averaged instantaneous firing rates for the visual (V, dark blue), auditory (A, dark red), and visual-auditory (VA, magenta) responses in the neurotypic cohort. All responses were aligned based on the Estimated Time of Convergence (ETOC) of the V and A inputs. A robust period of response enhancement was evident after the ETOC (the Initial Response Enhancement, IRE) as is typical of normally reared animals. **(B)** In the dark-reared cohort, VA responses were marginally significantly enhanced in the early window. This was followed by a period of significant response suppression. **(C)** A bar histogram summarizes the results. ***p < 0.001. Error bars show SEM.

However, the scaling of the multisensory transform in the dark-reared group was anomalous (Figure 1). Neurotypic SC neurons almost always show a robust and superadditive level of enhancement near the beginning of the multisensory response. This initial response enhancement (IRE) occurs when a neuron’s unisensory inputs first converge near the ETOC (Rowland et al., 2007), and can be measured in an early temporal window from (ETOC-20 ms) to (ETOC + 30 ms) (Miller et al., 2017). This finding was replicated in the neurotypic sample, in which VA responses were significantly enhanced within the IRE (ME = 96 ± 111%, 1-sample *t*-test, *p* < 0.001). Outside of the IRE, and in agreement with prior observations, neurotypic VA responses showed a decreased, but still significant, level of enhancement (ME = 39 ± 54%, 1-sample *t*-test, *p* < 0.001). Dark-reared neurons did not show this characteristic pattern (Figure 1B). Within the window defining the IRE response enhancement was far more modest (ME = 26 ± 31%) and in most (80%) neurons it was not significant, but did reach significance at the population level (1-sample *t*-test, *p* < 0.001). Outside the IRE, these neurons showed response suppression (ME = −8 ± 22%, 1-sample *t*-test, *p* < 0.001). These differences are summarized in Figure 1C.

To characterize the multisensory computations engaged, data from both populations of neurons were compared to the predictions of a model of statistical facilitation. This model makes the assumption that, at each moment in time, the multisensory response is determined by whichever input modality is stronger (but there is no interaction between them). Because responses show substantial inter- and intra-trial variation, the identity of the stronger input modality can change from trial to trial and also millisecond-by-millisecond within the same trial.

**FIGURE 2:**
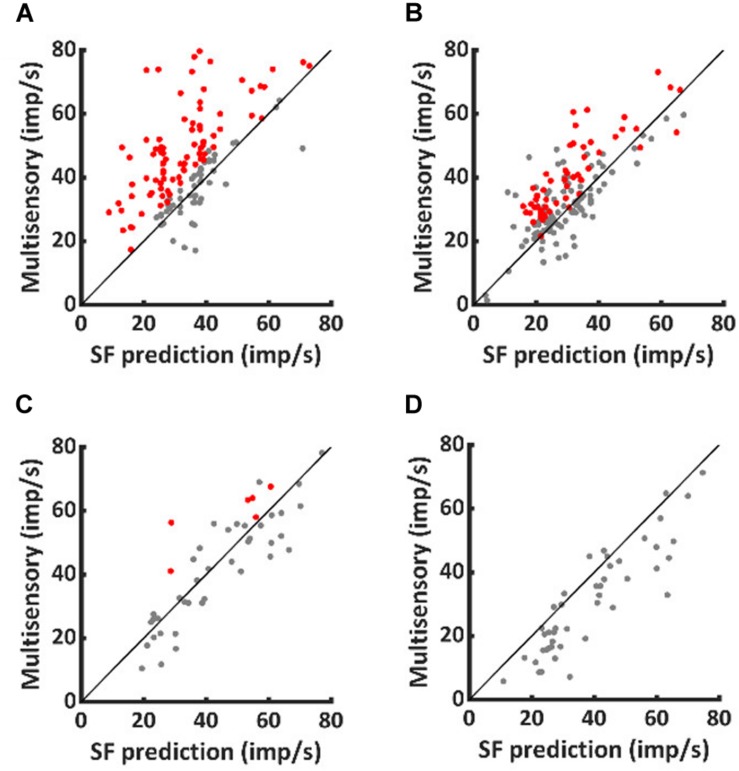
Normally-reared neurons commonly exceeded predictions of statistical facilitation, but only rarely did dark-reared neurons. Red dots identify the neurotypic neurons that exceeded statistical facilitation within **(A)** and (less often) outside **(B)** the IRE. Few comparable examples were obtained from dark-reared neurons within **(C)** or outside **(D)** the IRE.

As shown in Figure 2, VA responses in the neurotypic cohort exceeded the predictions of statistical facilitation by 31.4 ± 34.0% on average (1-sample *t*-test, *p* < 0.001). The enhancement above statistical facilitation was prominent within the IRE (56.7 ± 66.0%, 1-sample *t*-test, *p* < 0.001, Figure 2A), but was not significantly different from statistical facilitation outside the IRE, despite being numerically larger (20 ± 37.7%, 1-sample *t*-test, *p* = 0.08, Figure 2B). Notably, one or both of the unisensory responses were in significant decline beyond the IRE; thus, input magnitude was substantially reduced.

A very different pattern was evident in the dark-reared cohort. Averaged over the entire response window, VA responses were suppressed relative to statistical facilitation (−18 ± 20%, 1-sample *t*-test, *p* < 0.001). Their responses were consistent with statistical facilitation within the IRE (−2 ± 29%, 1-sample *t*-test, *p* = 0.79, Figure 2C) and significantly below statistical facilitation outside it (−23 ± 21%, 1-sample *t*-test, *p* < 0.001, Figure 2D).

Although these data suggest that the difference between normal and dark-reared multisensory response capabilities are in the multisensory transform itself, there are several other unisensory properties that have been shown to be capable of influencing multisensory responses.

### Unisensory Response Magnitude and Balance

**FIGURE 3:**
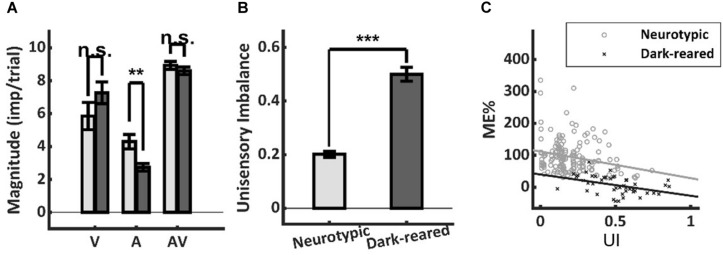
Response magnitude and unisensory imbalance. **(A)** Average visual (V) and multisensory (AV) responses were not significantly different in normal and dark-reared neurons (albeit dark-reared auditory responses were weaker). **(B)** There was significantly more unisensory imbalance (UI) in the dark-reared group. **(C)** However, the regressions of ME vs. UI showed significantly different intercepts: dark-reared animals show a defect even after controlling for UI. Lines represent the least-squared fits. **p < 0.01; ***p < 0.001. Error bars show SEM.

It is well-established that more robust unisensory responses in the SC are associated with smaller proportionate multisensory enhancements (Meredith and Stein, 1986b; Stein et al., 2009; Ohshiro et al., 2011; Otto et al., 2013; Truszkowski et al., 2017). Thus, it was possible that low ME in the dark-reared group could reflect more robust unisensory responses. However, the visual responses of the two groups were not significantly different (dark = 7.26 ± 3.94 imp/trial, neurotypic = 5.85 ± 6.95 imp/trial, *p* = 0.26), and the auditory responses of the dark-reared group were weaker on average (dark = 2.74 ± 1.52 imp/trial, neurotypic = 4.30 ± 3.04 imp/trial, *t*-test, *p* = 0.003) (Figure 3A). If the multisensory transform were equivalent, this would have led to an equal or higher ME in the dark-reared than in the neurotypic population.

Lower ME scores are also associated with large imbalances between the unisensory responses in a sample (Otto et al., 2013; Miller et al., 2015). And the dark-reared sample was found to be heavily visual-dominant (97 vs. 73% for neurotypic) with correspondingly higher levels of imbalance (dark UI = 0.50 ± 0.17 vs. neurotypic UI = 0.20 ± 0.14, *p* < 0.001) (Figure 3B). Yet this factor could not explain the lack of enhancement in the dark-reared group. ME was inversely related to UI within each cohort (dark-reared: *R*^2^ = 0.17, *p* < 0.01, neurotypic: *R*^2^ = 0.06, *p* < 0.01), and the slopes of these relationships were not significantly different (dark: −0.67, neurotypic: −0.84, *t*-test, *p* = 0.72) (Figure 3C). But, there were substantial differences in the intercepts of these regressions (dark: 40%, neurotypic: 112%, *t*-test, *p* < 0.01). Thus, although the dark-reared group shows greater imbalance, the neurotypic group shows much greater ME scores (∼3X) even after controlling for this factor: there is a significant decrease in ME in the dark-reared group observed at all levels of UI.

### Unisensory Temporal Alignment

**FIGURE 4:**
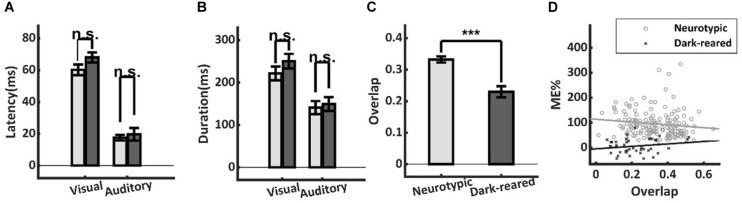
Temporal differences in neurotypic and dark-reared cohorts. **(A,B)** There were no significant differences in the visual or auditory onset latencies or response durations. **(C)** Calculated on a sample-by-sample basis there was lower temporal overlap between the unisensory inputs in the dark-reared cohort. **(D)** However, regression models for neither cohort yielded slopes that were significantly different from zero. Thus, differences in temporal overlap did not explain their ME differences. ***p < 0.001. Error bars show SEM.

Temporal misalignment in the cross-modal inputs to a neuron can also substantially reduce multisensory enhancement (Meredith et al., 1987). However, this proved not to be a significant factor here: there were neither significant differences in the onset latencies of the visual (dark: 68.0 ± 18.1 ms, neurotypic: 60.2 ± 28.3 ms, *t*-test, *p* = 0.16) or auditory (dark: 19.7 ± 22.5 ms, neurotypic: 17.6 ± 11.8 ms, *t*-test, *p* = 0.58) responses of normal and dark-reared animals (Figure 4A), nor in their response durations (Visual: dark-reared: 250.4 ± 114.7 ms, neurotypic: 221.6 ± 135.3 ms, *t*-test, *p* = 0.24; auditory: dark: 149.2 ± 110.0 ms, neurotypic: 141.0 ± 106.3 ms, *t*-test, *p* = 0.72) (Figure 4B).

A lack of temporal overlap between the cross-modal inputs could reduce ME in principle. And indeed, there was slightly lower overlap of the unisensory inputs in the dark-reared group (dark = 23 ± 0.12%, neurotypic = 33 ± 0.12%, *t*-test, *p* < 0.001) (Figure 4C). However, regressing ME against temporal overlap failed to show a significant slope in either case (dark-reared: *R*^2^ = 0.018, *p* = 0.09, neurotypic: *R*^2^ = 0.043, *p* = 0.49) and the intercepts differed significantly (dark: −5%, neurotypic: 114%, *t*-test, *p* < 0.001) (Figure 4D). Thus, the difference in ME scores remained even after controlling for differences in the temporal overlap between the two groups.

In sum, neither differences in unisensory magnitudes nor temporal dynamics could explain the differences between normal and dark-reared multisensory responses. In contrast, there were categorical differences in the multisensory transform in all phases of their responses. The neurotypic multisensory response showed a characteristic shift from a period in which the computation was superadditive (within the IRE) to a trailing period in which the computation was consistent with statistical facilitation. In contrast, the dark-reared response computation was initially consistent with statistical facilitation, and then shifted to one that yielded response suppression.

## Discussion

Depriving animals of unisensory (e.g., visual) experience disrupts their multisensory development. They fail to craft the ability to properly synthesize its inputs with those from other modalities (see review by Stein et al., 2014). These defects persist even when later experience is available in a normal housing environment (Xu et al., 2017) and resemble, in a general sense, the multisensory processing abnormalities observed in a number of human psychiatric populations (Brett-Green et al., 2010; Williams et al., 2010; Brandwein et al., 2013; Stevenson et al., 2014, 2017; Beker et al., 2018). There is significant interest in understanding the mechanisms operating in these defective states. Recent work has demonstrated that, at a macroscopic level, multisensory processing in the naïve state reflects a competitive, rather than a cooperative, interaction among the senses. Thus, the “default” multisensory computation fails to yield an enhanced response, and often yields one that is lower than the most effective of its unisensory component responses (Yu et al., 2018).

The present study shows that the multisensory responses of dark-reared neurons are anomalous throughout their entire time course: they do not show the characteristic enhancement early in the response, and show response suppression in later phases. Thus, the deficit is not explained by atypical unisensory inputs despite minor alterations in their magnitudes and timing. However, these differences suggest that one of the consequences of multisensory experience may be the calibration of these input features onto common multisensory neuron targets. Such calibration could be produced by Hebbian algorithms speculated to operate in this circuit (Cuppini et al., 2012, 2018; Yu et al., 2019). Briefly, repeated bouts of temporally overlapping activity among cross-modal presynaptic inputs, coupled with post-synaptic activation, should selectively strengthen inputs with congruent temporal properties. If the strengthening is inversely proportional to the baseline synaptic strength (Cuppini et al., 2018), then effective cross-modal inputs which repeatedly activate in tandem will eventually become equally-strong.

The defects observed in multisensory enhancement were mostly attributable to a defect in the moment-by-moment multisensory transform. Recent work suggests that the neurotypic transform can be explained by a simple mechanistic model in which cross-modal input currents sum linearly and multisensory responses engage an additional delayed, calibrating inhibition (Miller et al., 2017). Because the generation of action potentials is inherently non-linear (Rowland and Stein, 2014), this results in the characteristic superadditive IRE that is rescaled to a linear operation by the calibrating inhibition, presumably representing an inhibitory network or intrinsic dynamics that are offsetting of the large amplifications seen early in the response. The abnormal multisensory transform observed here indicates the operation of a different functional architecture, as proposed by Yu et al. (2018). The initial interaction in the dark-reared multisensory SC neuron is consistent with statistical facilitation (i.e., suppression of the weaker input) rather than linear current summation, and the following response is consistent with suppression. This pattern of interaction could be supported by an input configuration that is initially competitive, rather than cooperative. In such a scenario, SC afferents produce both excitatory influences on target SC neurons and inhibitory influences that strongly suppress inputs derived from other modalities (Cuppini et al., 2012, 2018; Yu et al., 2018).

Although the analyses here focus on the dark-reared neuron as a model of multisensory dysfunction following deprivation of multisensory experience during development, these findings likely extend to other populations. Prior work has demonstrated impairments in multisensory enhancement consequent to rearing animals in omnidirectional masking noise (Xu et al., 2014, 2017) as well as with visual and auditory stimuli that are presented with randomized spatial and temporal relationships (Xu et al., 2012). In addition, similar defects have been observed when crucial cortico-collicular inputs derived from association cortex are deactivated during early life when multisensory integration capabilities are typically developing (Rowland et al., 2014). We predict that, in each of these cases, the multisensory transform by which visual and auditory inputs are integrated to yield a cooperative interaction will also fail to develop, resulting in the retention of a maladaptive default competition.

How these findings ultimately relate to the human developmental and psychiatric cohorts identified above remains to be determined. These human conditions involve substantial cognitive abnormalities beyond multisensory integration, and have been associated with a variety of systemic issues ranging from synaptic anomalies to macrostructural changes in large-scale neuronal networks. Any and all of these changes could conceivably affect the multisensory transform directly and/or indirectly. However, the similarities in the multisensory defects in these human populations and the animal model suggest some common causality. In this context it may be helpful to consider that the effectiveness of multisensory training paradigms in changing both unisensory (Yu et al., 2009, 2013a) and multisensory (Yu et al., 2010, 2013b, 2018; Xu et al., 2017) processing dynamics might provide therapeutic possibilities for ameliorating this particular dysfunction. The present results suggest that anomalous early life experience can lead to anomalous multisensory processing by changing the way that modality-specific signals are transformed by multisensory neurons into an integrated product. Thus, strategies targeted on altering or shaping this transform are likely to be of substantial value.

## Data Availability Statement

The datasets generated for this study are available on request to the corresponding author.

## Ethics Statement

The animal study was reviewed and approved by Animal Care and Use Committee of Wake Forest Medical School.

## Author Contributions

ZW performed the analysis and wrote the manuscript. LY and JX designed the research, collected the data, and performed the analysis, and wrote the manuscript. BS and BR designed the research, performed the analysis, and wrote the manuscript.

## Conflict of Interest

The authors declare that the research was conducted in the absence of any commercial or financial relationships that could be construed as a potential conflict of interest.
